# The Impact of COVID-19 on Well-Being: Welsh Children’s Perspectives

**DOI:** 10.1007/s42448-023-00149-w

**Published:** 2023-03-20

**Authors:** Jennifer Hampton, Colette McAuley

**Affiliations:** 1 Wales Institute of Social and Economic Research, Data and Methods, Cardiff University, Cardiff, Wales; 2 CASCADE Research Centre, Cardiff University, Cardiff, Wales

**Keywords:** Wales, COVID-19, Children, Well-being

## Abstract

The COVID-19 pandemic saw drastic and unprecedented actions by governments to mitigate the spread of the virus. Often, the restrictions limited in-person interaction and included the closure of schools. To investigate the impact of both the pandemic and resulting restrictions, the International Society of Child Indicators developed the Childrens Worlds: COVID-19 Supplement. This paper reports on the results of that survey in Wales in 2021. Seven hundred and twenty seven children from 18 schools participated from years 6 and 8. They received an anonymous survey asking about their circumstances and well-being across a range of domains, and how these have changes during the pandemic. The children had experienced significant changes in their lives with the onset of the pandemic. The majority could not attend school, were confined to their homes, and were unable to see wider family and friends in person. Almost a half of both groups felt that their relationships with family they lived with had improved, with many becoming closer to members. Over one-fifth of both groups thought their relationships with friends were affected, with younger children more likely to think they had improved. The pattern throughout the survey was that the older children were less positive in their responses. The disparity between the groups was markedly so regarding school with the secondary schoolchildren being particularly dissatisfied with the content of their learning. Whereas there was a trend for less disparity between the groups during COVID-19, the only area where the disparity increased was regarding satisfaction with school. These findings are then placed in the context of developments in education in Wales and research on the impact of COVID on Welsh schools and schoolchildren. As in other countries, the pandemic would appear to have exacerbated existing educational inequalities.

## Introduction

Wales is one of the four nations of the UK with its own devolved government since 1999. It makes up 8.4% of the UK’s landmass, at just under 21,000 square kilometres. The latest figures indicate that the population of Wales at the time of the 2021 Census was 3,107,500 (ONS, 2022). Of these, 16.5% (513,800) were aged under 15 years. It is important to mention the bilingual nature of Wales, with nearly a third (29.9%) of the population able to speak some Welsh. Between a third and a quarter of all primary and secondary schoolchildren learn through the medium of Welsh. The Welsh government is clearly committed to enhancing the well-being of its child and adult citizens, as evidenced in the Social Services and Well-Being (Wales) Act 2014 which came into force in 2016.

The Welsh Institute of Social and Economic Research and Data (WISERD) undertook the first *Children’s Worlds* survey in Wales in 2018. This is a research survey on children’s subjective well-being developed by the International Society of Child Indicators (ISCI). This was the first such survey in Wales and also permitted comparison with other participating countries. In Wales, 959 ten-year-olds from 34 primary schools and 1668 twelve-year-olds from 20 secondary schools participated. Two overall findings were particularly notable. Compared to other countries, Welsh children’s subjective well-being appeared to be relatively low. Secondly, the area which showed most variability, and reflects some of the highest areas of children’s dissatisfaction, appeared to be their experience of school. This was markedly so for the older children at secondary schools.

The COVID-19 pandemic saw drastic and unprecedented actions to mitigate the spread of the virus. Many countries applied restrictions and guidelines that limited in-person interaction between individuals, including the closure of schools to the majority of children. As a result, ISCI developed a further survey entitled *Children’s Worlds: Covid-19 Supplement* to investigate the impact of the pandemic and resultant social restrictions on children’s well-being. Over 20 countries took part, including Wales.

This paper is based upon the initial analyses of the *COVID-19 Supplement* survey carried out in Wales in 2021, with 727 children participating from 18 primary and secondary schools.

## Children’s Subjective Well-Being

Child well-being as a concept has been the subject of increasing interest and usage over the past three decades (McAuley & Rose, 2010). Measuring and monitoring well-being became a key field of academic interest called the Child Indicator Movement. Ben–Arieh (2005) has argued that both normative changes and methodological advances acted as driving forces. The United Nations Convention on the Rights of the Child (United Nations, 1989) emphasised the right of the child to have a voice in matters affecting them. The Sociology of Childhood introduced the notion of children as social actors interacting and actively contributing to their own environments. Widespread acceptance of the Bronfenbrenner’s ecological model of development emphasised the need to consider all aspects of a child’s and in the context of family, community and wider environment. This model proposes that children constantly interact with the environment by balancing factors, using resources and responding to stress (Bronfenbrenner, 1979; Bronfenbrenner & Morris, 2006).

On the methodological side, it became clear that objective measures needed to be complemented by subjective perspectives of childhood (Casas, 2011). Hence, the child indicator movement sought child-centred indicators. As the child became the unit of observation, new domains emerged. Interest arose in children’s experience in the here and now and consequently their daily lives, relationships and views and feelings became the subject of attention.

Incorporating children’s subjective well-being became both a prerequisite and a consequence of the new field of measuring and monitoring well-being (Ben-Arieh, 2008). Although concerns were initially raised about the reliability and validity of children’s accounts, it is now well accepted that to develop our understanding of child well-being, we need to ask children directly (Ben-Arieh, 2010). Moreover, to understand their social and emotional relationships, the views of children are crucial (Ben-Arieh, 2008).

Researchers’ interest in children’s subjective well-being has been rapidly increasing for well over a decade (McAuley, 2012). It has garnered interest from both qualitative and quantitative researchers in the international community. Most notably, the International Society of Child Indicators (ISCI) has supported two global research initiatives: the *Children’s Worlds* survey (see www.isciweb.org) and the *Children’s Understanding of Well-Being: Global and Local Contexts* multinational qualitative study (see www.cuwb.org).

Children’s Worlds is a worldwide research survey on children’s subjective well-being. It aims to understand children’s subjective well-being and how they experience daily activities within their families, neighbourhoods and at school, using a quantitative survey of children across 24 nations. Results of the first two waves have been published (Dinisman et al., 2015; ISCIWeb, 2020). The third wave of the survey was completed in 2018, with 128,000 children from 35 countries taking part. Identified early on by Goswami (2012), children’s relationships are centrally important to their sense of well-being. A recent qualitative Children's Understanding of Well-Being study in England reinforced this finding and examined the children's rationalisations (McAuley 2019). 

## Children’s Worlds Survey in Wales

As indicated above, the devolved government in Wales has a clear commitment to enhancing the well-being of its child and adult citizens, as evidenced by its Social Services and Well-Being (Wales) Act 2014. Hence, the Welsh Institute of Social and Economic Research and Data (WISERD) undertook the first Children’s Worlds Survey in Wales in 2018 as part of the third wave. This provided a unique opportunity to gather information from Welsh children about their well-being and permitted comparison with children’s responses in other countries.

Results have been reported (Hampton et al., 2019). Of particular importance was the relatively low levels of well-being reported by children in Wales in comparison with other countries. Details about the findings from the third wave across multiple countries can be accessed at isciweb.org/the-data/wave-3/.

Secondly, in relation to the Welsh children, the area which showed most variability, and reflects some of the highest areas of children’s dissatisfaction, appeared to be their experience of school. This was markedly so for the older children at secondary schools.

## Children’s Worlds: Covid‐19 Supplement Survey in Wales 2021

As detailed earlier in the introduction, ISCI developed a further survey entitled the *Children’s Worlds: COVID‐19 Supplement* to investigate the impact of the pandemic and the resultant social restrictions on children’s well‐being. This was felt to be particularly important, given our increasing understanding of the importance of relationships to their wellbeing. Over 20 countries took part, including Wales. Here we report on the method and preliminary findings of the survey in Wales (Hampton, 2021).

### Method

#### Sampling

Sampling for the survey was conducted via schools. The schools approached had participated in Wave 3 of the Children’s Worlds survey (Hampton et al., 2019), with original schools drawn from a stratified random sample based on socioeconomic characteristics of their local areas. Schools were approached to participate in this supplementary survey via email, using either established contacts within the school, the headteacher, or general email address. They were asked to distribute their unique link to their pupils in year 6 (primary schools) or year 8 (secondary schools) via email. Of the schools approached, 18 schools participated (11 primary and 7 secondary) with 727 children responding to the survey (293 primary school pupils, 434 secondary school pupils). The survey was completed towards the end of the school year (the latter half of the summer term), with average ages reflecting the older end of these year groups (11-years-old and 13-years-old, for primary and secondary school pupils, respectively). Data were collected in 2021, with pupils having experienced multiple periods of lockdown and school closures in Wales.

#### Survey Design

The survey was designed in collaboration with the wider Children’s Worlds team, which consists of researchers experienced in this type of research from a wide range of countries (for more information see https://isciweb.org). An original draft was created by the central team with translation and piloting for comprehension conducted by local national teams. In Wales, the survey was offered in both Welsh and English. The survey consisted of several areas of interest including topics related specifically to the COVID-19 context and their experiences of this, as well as more general questions about how they feel about their lives. The purpose of the survey was to gather information about children’s lived experience of the COVID-19 pandemic, what restrictions they were under, and how they felt these restrictions and the situation had impacted on their daily activities, relationships, and overall well-being. The questions were presented as a series of agreement statements and closed multiple-choice questions.

#### Data Collection and Analysis

All data collection was conducted online via Qualtrics, which allowed for convenient and anonymous responses to be submitted. Although individuals remained anonymous, in that no identifying information about the participants was requested nor retained, schools were given unique links so that analysis could be conducted at a school level for reporting purposes. Furthermore, this approach allowed for the possibility of examination of school-level effects. Analysis presented in this paper is descriptive and was conducted in R.

### Ethics

The study was approved by Cardiff University Social Sciences Ethics Committee.

### Findings

The results are presented in the order in which the questions were presented to the children in the survey questionnaire.

### What the Children Told Us About Themselves

The majority of the primary (PS) and secondary (SS) schoolchildren reported that they could not attend school and had to stay at home for many days. They indicated that everyone in town was in lockdown for many days. Over half of the primary schoolchildren and almost two-thirds of the secondary schoolchildren knew someone outside their family who got infected with COVID-19.

### Their Personal Situation During COVID-19

The vast majority (89.3% PS and 93.5% SS) of both groups reported that their school was closed during lockdown. Again, the majority (76% PS and 81.1% SS) had to be in their homes all day. Almost three-quarters of the secondary schoolchildren indicated that they were only allowed at times to leave home for a few hours during the day. Considerably fewer but still over half (57.4%) of the primary schoolchildren also reported this.

The majority of both groups (68.5% PS and 70.9% SS) agreed with the statement that they felt safe at home. Over half of each group (53.9% and 53.4%) reported feeling safe with friends. Notably, more primary schoolchildren agreed with the statement that they felt safe at school than secondary schoolchildren (42% PS and 21.5% SS).

Both groups reported missing relatives, with primary schoolchildren agreeing with this statement more often (55.8% PS and 45.7% SS). Again, primary schoolchildren reported missing friends more often (45.9% PS and 28.7% SS).

### Life During COVID-19

The predominant source of information about COVID-19 for all the children was the news (50.7% PS and 44.9% SS). The next most popular source was their family (26.2% PS and 26.7% SS). Slightly more of each group stated they had sufficient COVID information (29.6 PS and 27% SS).

### Satisfaction with Lives Pre-COVID and During COVID

When asked about satisfaction with life pre-COVID, the pattern with the primary schoolchildren was of relatively higher satisfaction with people they lived with, relationships with friends, things they used to learn at school, and how they spent their time. Secondary schoolchildren reported somewhat *lower* satisfaction on all of these aspects of their lives, but markedly so on things they learnt at school (8.8% PS and 15.9% SS).

The children were then asked the same questions about their lives during COVID. Again, the pattern was of primary schoolchildren being relatively more satisfied on all the same aspects. Secondary schoolchildren reported somewhat *lower* satisfaction on all of these aspects of their lives but again markedly so on things they learnt at school (22.8% PS and 36.2% SS).

An examination of the means for both groups compared at both time points (pre- and during COVID) suggests that there was less disparity between the groups on how they spent their time and the relationships with people they lived with apart from on the issue of the things they learnt at school. In this case, the disparity in level of satisfaction increased during COVID, with secondary schoolchildren reporting much lower satisfaction.

### How Children Spent Daily Time During COVID

Children were asked how many times a week they spent their time doing a range of activities. The most often reported for both groups in order were using social media, watching moves or TV series, playing games on the computer, mobile phone or devices, speaking on the phone or video call, playing or hanging out inside the house, and spending time on own. The largest disparity between the two groups was on using social media (52.9% PS and 73.5% SS). Over half of both groups reported watching movies or TV series (53.6% PS and 53.5% SS).

In response to a series of further questions on which their agreement was sought, there was considerable agreement from both groups that they could spend more time with their family (46.9% PS and 40% SS). Fairly similar agreement from both groups that they could sleep longer (29.6PS and 28.6% SS) and could make their own time schedule (21.3% PS and 23.2% SS). However, there was much higher relative agreement by primary schoolchildren on learning new methods of doing schoolwork over web (35.3% PS and 23.6% SS). In response to a further question about if they managed to continue with learning from home during COVID when schools were closed, a much higher percentage of primary schoolchildren agreed with this statement (45.2% PS and 25.4% SS).

### Children’s Relationships and School During COVID

Both groups confirmed a very high rate an access to internet during this period (71% PS and 88% SS). Over half (52.2%) of the primary schoolchildren missed their classmates, and over a third (36.5%) wished they could go back to school. This compared with 29.4% and 17.4% respectively of secondary schoolchildren. More primary schoolchildren reported help with homework from parent or sibling (22.8% PS and 17.6% SS) and that they missed their teacher’s advice (14.3% PS and 9.8% SS). A smaller but more similar percentage of both groups reported having problems with the internet during web classes (6.1% PS and 4.8% SS) and not being able to access the internet for an entire day (5.7% PS and 6.0% SS). More (33.2%) secondary schoolchildren viewed themselves as high users of social network/media compared to 27.8% primary schoolchildren. Whilst over two-thirds (68.6%) of primary schoolchildren reported managing school platforms quite easily, less (64.3%) of secondary schoolchildren did.

In terms of statements about those they felt well supported by, the majority (63.8%) of primary schoolchildren selected the people they lived with, 29.2% indicated some of their teachers, and 24.4% chose some of their friends. This compared with respectively 53.9%, 13.3% and 23.4% by the secondary schoolchildren. The former were much more likely to indicate support from those they live with and some of their teachers.

Over one-fifth of both groups (21.7% PS and 22.4% SS) thought that their relationships with friends had been affected by the Coronavirus. Over a third of both groups (35.1% PS and 35.3% SS) thought they had become closer to some members of their family during this time.

In terms of other relationships during the pandemic, around a quarter of the primary schoolchildren thought they were better with friends who lived nearby, friends from school, and family who lived elsewhere, whereas somewhat less secondary schoolchildren did. However, almost one-half of both groups (47.7% PS and 49.7% SS) thought that relationships with family they lived with were better.

More primary schoolchildren agreed that they make decisions about their lives together with their parents (43.2%); their parents listen to them and take what they say into account (54.5%) and have a good time together in their families (62.0%); people in their family would help them with problems (58.7%) and care about them (76.3%). Secondary schoolchildren were less in agreement on all of these aspects, scoring respectively 34.9%, 46.6%, 52.8%, 55.6%, and 68.2%.

When asked about school relationships, there was considerable disparity between the two groups regarding having opportunities at school to make decisions important to them (41.7% PS and 19.8% SS), teachers listening to them and taking what they say into account 39.1% PS and 15.7% SS), if they have a problem at school whether other children (30.4% PS and 15.8% SS) or their teachers (49.3% PS and 16.2% SS) will help them, and if their teachers care about them (46.9% PS and 13.7% SS). The only area where there were similar levels of agreement was on the statement that there are a lot of arguments between children in their class (20.6% PS and 17.1% SS).

In answer to statements concerning friendships, more primary schoolchildren agreed about having a friend to support them if they have a problem (55.2% PS and 49.7% SS), getting along well with friends (51.9% PS and 47.3% SS), friends usually being nice to them (49.4% PS and 41.9% SS), and having enough friends (50.6% PS and 45.8% SS). Whilst almost half of the children agreed to these statements, notably less of the secondary schoolchildren agreed that friends are usually nice to them.

The children were asked about being left out by other children in their class, called unkind names or hit by children from their school. Both groups reported similarly that they had never been called unkind names 45% PS and 43.5% SS) or hit (56.6% PS and 57.7%) by other children from their schools. There was a greater disparity in reports from both groups concerning being left out by other children from their class (42.8% PS and 56.1% SS).

### How the Children Feel About Their Lives

Overall, primary schoolchildren reported feeling more positive on all statements concerning their lives. This included feeling positive about their futures, feeling that they are learning a lot at the moment, have enough choice about how they spend their time, people being generally friendly towards them, being good at managing their daily responsibilities, liking the way they are, being happy with their lives, thinking that the things that happen in their lives are excellent, thinking they have a good life, thinking their life is going well, and enjoying their life. The largest disparities in *low agreement* were on the statements ‘I feel positive about my future’ (8.3% PS and 19.53% SS); ‘I feel that I am learning a lot’ (9.6% PS and 21.28% SS); ‘I am good at managing my daily responsibilities’ (7.3% PS and 20.37% SS); and ‘I like being the way I am’ (12.3% PS and 23.54% SS).

They were asked how much they had felt this way in the past two weeks. This included feeling bored, full of energy, stressed, calm, sad, or happy. A mixed picture emerges here, with the majority of children reporting feeling at least somewhat happy (91.6% PS, 87% SS) and calm (80% PS, 72.1% SS). However, over half were also likely to report feeling at least somewhat stressed (53.2%, 61% SS). Although mainly reporting similar patterns, differences were indicated between the ages when it came to feeling full of energy and bored; on average, younger children were more likely to report feeling full of energy (6.23/100) and, conversely, less bored (4.97/10) than their older counterparts (5.85 and 5.39 respectively).

The children were also asked about the impact of the virus on them personally which included statements about whether their heart raced when thinking about getting the virus, being unable to sleep worrying about getting it, becoming nervous or anxious when watching news and stories about the virus, being afraid of losing their lives due to the virus, hands becoming sweaty when thinking about it, feeling uncomfortable when thinking about it, and feeling very afraid of the virus. Overall, agreement with these statements was low for both groups. However, primary schoolchildren had somewhat higher levels of agreement on all the statements. On two statements ‘I am afraid of losing my life because of the Coronavirus’ (12.4%) and ‘I am very afraid of the Coronavirus’ (12.7%), primary schoolchildren were much more likely to agree.

When the children were asked about their level of satisfaction with their lives, the secondary schoolchildren were less satisfied on all statements. These statements included life as a whole, health, being listened to by adults, what may happen later in their lives, the freedom they have, the things they have, the way they look, how they use their time, their friends, how safe they feel, the area where they live, the house they live in, and the people they live with. There was considerable disparity on the majority of statements between the two groups. Most notably, over one-third (35.1%) of secondary schoolchildren indicated low satisfaction with the way they looked in comparison to 19.8% of primary schoolchildren. Other areas of consider disparity in order were their low satisfaction with how they are listened to by adults, their health, what may happen later in life, and life as a whole.

Perhaps unsurprisingly, there appears to have been an increase in dissatisfaction with the onset of the pandemic, with children reporting lower levels of satisfaction with their lives as a whole during the pandemic, compared to levels of satisfaction before this. Although more children reported being not at all or only a little happy with their lives (PS 17.3% during, 6.4% before, SS 23.1% during, 10.5% before), the vast majority were at least somewhat satisfied at both time points, with averages in the upper half of the scale both before (PS 7.22, SS 6.84) and during the pandemic (PS 6.25, SS 5.98).

Children were asked the extent to which they agreed with a number of statements about the detail of their lives, including positivity about the future, amount of learning, opportunity for choice about time use, the friendliness of others, responsibilities, and how much they liked the way they are. Although the majority responded at least somewhat positively to these statements, there were notable differences between the age groups, with older children more likely to respond with little or no agreement. The largest differences can be seen with older children feeling less likely that they were learning a lot during the pandemic (on average, PS 6.77/10, SS 6.00/10) or feeling positive about the future (PS 7.14, SS 6.46).

Finally, children were asked about how important it was to find a solution to three areas: the coronavirus, their own feelings of safety, and their families’ money problems. The most important of these for children who responded was finding a solution to the coronavirus, with over 80% across the ages considering this at least somewhat important. Although considered less important, differences were apparent between the age groups when it came to finding a solution to their own feelings of safety, with younger children considering this more important than older children (little or not at all PS 31.6% SS 43.6%).

## Discussion/Conclusion

Perhaps the most striking finding from the COVID-19 Supplement survey was the level of some children’s dissatisfaction with schools and learning content, particularly among the secondary school cohort. Clearly there was dissatisfaction pre-COVID, but this was exacerbated during COVID and particularly so for the older group. This is consistent with the findings of the earlier Welsh Children’s World Study. So what do we know about education in Wales and why might the impact of COVID affect children differently?

Wales has been involved in recent years in large-scale school improvement reform to develop a high performing educational system characterized by quality and equity. However, useful context is provided by Foster (2021) who indicates that around 29% children in Wales live in relative income poverty and approximately 18% of children aged 5 − 18 years are entitled to free school meals. There already was an educational attainment gap estimated at 16% at age 11 years increasing to 32% by GCSE level (Joseph Rowntree Foundation, 2018). Reducing that gap was an area of focus for the Welsh government prior to the pandemic (Welsh Government, 2017).

In terms of the rapid adjustment of schools and teachers to working during the pandemic, Waters-Davies et al. (2022) concluded that that it was a period of learning for all involved in education. Foster (2021) indicates that blended learning was not the norm in Wales before the pandemic so embedding infrastructure and support would take time to develop fully.

Several other studies and inspections have specifically considered the impact of COVID and the lockdown on schoolchildren in Wales. Taylor (2020) surveyed 560 children in secondary schools in Wales during the first lockdown experience. He found a significant disparity in number of hours children spent on schoolwork at home during lockdown, with around forty per cent indicating that they spent less than or around 10 h per week whilst over 15% spent twice that time per week. Children relied on teachers about what to study and their main form of contact was via email with little or no direct contact. Half of the children rarely or never shared homework or received help from parents. Further, the Estyn December 2021 School Inspectors Report found considerable variation in the online provision offered by schools. In particular, the use of live lessons varied greatly. They also found that children with additional learning needs engaged less with online learning. When school re-opened, there was lower attendance, particularly of children from disadvantaged backgrounds.

Hampton (2020) also raises the issue of potential unequal consequences that lockdown, especially school closure, had on children from different backgrounds. She argues that the lack of a suitable place to study may be a significant factor for children from more disadvantaged backgrounds. Foster (2021) also states that whilst the Welsh government provided funding to ensure digital inclusion during this period, the lack of a suitable home learning environment may well have contributed to widening inequalities. She also suggests that children with special needs and/or living in difficult home circumstances may not have received adequate support.

In summary, it would seem that the variable level of online provision, the lack of a suitable home learning environment and children with additional learning or support needs finding it difficult to engage with online resources have all been identified as important factors. These may well have contributed to some children feeling dissatisfied with school and the content of their learning. The importance of the wider context is also clear. The latest Estyn School Inspectors Report (September 2022) concluded that the pandemic hit children from disadvantaged backgrounds hardest. In fact, it is estimated that the pandemic has exacerbated existing educational inequalities worldwide (United Nations, 2020). In any study of well-being, context remains a crucial consideration (Fattore et al, 2019).

## References

[CR1] Ben-Arieh A (2005). Where are the children? Children’s role in measuring and monitoring their well-being. Social Indicators.

[CR2] Ben-Arieh A (2008). The child indicators movement: Past, present and future. Child Indicators Research.

[CR3] Ben-Arieh A, McAuley C, Rose W (2010). Developing indicators for child well-being in a changing context. Child Well-Being Understanding Children’s Lives.

[CR4] Bronfenbrenner U (1979). The Ecology of Human Development: Experiment by Nature and Design.

[CR5] Bronfenbrenner U, Morris P, Damon W, Lerner R (2006). The bioecological model of human development. Handbook of Child Psychology (Sixth Edition).

[CR6] Casas, F. (2011). Subjective social indicators and child and adolescent well‐being. *Child Indicators Research,**4*(4), 555–575.

[CR7] Dinisman T, Fernandes L, Main G (2015). Findings from the first wave of the ISCWeb project: International perspectives on children’s subjective well-being. Child Indicators Research.

[CR8] Esytn School Inspection Reports (September 2022). Available from https://www.estyn.gov.wales. Accessed 20 Dec 2022.

[CR9] Fattore T, Fegter S, Hunner-Kreisel C (2019). Children’s understanding of well-being in global and local contexts: Theoretical and methodological considerations for a multinational qualitative study. Child Indicators Research.

[CR10] Foster C (2021). The education response to coronavirus: Implications for schools in Wales.

[CR11] Goswami, H. (2012). Social relationships and children’s subjective well-being. *Social Indicators Research,* *107, *575–588.

[CR12] Hampton, J. M. (2020). *Covid-19, home learning and educational inequalities*. Wales Institute of Social and Economic Research News. Accesssed 2 Sept 2022.

[CR13] Hampton JM (2021). Children’s worlds: Covid supplement Wales.

[CR14] Hampton, J. M., Power, S. and Taylor, C. (2019) Children’s World National Report WALES. Wales: Wales Institute of Social and Economic Research. Available at https://isciweb.org/wp-content/uploads/2020/03/Wales-National:Report-Wave-3.pdf

[CR15] ISCIWeB (2020) Children’s views on their lives and well—Being in 35 countries: A report on the Childrens Worlds Project, 2016–2019. Available at www.isciweb.org.

[CR16] Joseph Rowntree Foundation. (2018). Education in Wales. Retrieved from https://www.jrf.org.uk/data/education-wales. Accessed 5 Sept 2022.

[CR17] McAuley C, Rose W (2010). Child well-being: Understanding children’s lives.

[CR18] McAuley C (2012). International advances in child well-being: Measuring and monitoring subjective well-being. Child Indicators Research.

[CR19] McAuley C (2019). Exploring eleven year old children’s understanding of well-being using well-being maps: Commonalities and divergences across areas of varying levels of deprivation and diversity in an English qualitative study’. Children and Youth Services Review.

[CR20] Office of National Statistics (ONS). (2022). Census 2021: Data and Analysis from Census 2021 England and Wales. Retrieved from https://www.ons.gov.uk. Accessed 3 Nov 2022.

[CR21] Taylor C (2020). The impact of Covid-19 on Children’s learning in Wales.

[CR22] United Nations. (1989). *UN Convention on the Rights of the Child*. Geneva: United Nations.

[CR23] United Nations. (2020). *The sustainable development goals report.* Retrieved from https://unstats.un.org/sdgs/report/2020/The-Sustainable-Development-Goals-Report-2020.pdf. Accessed 2 Sept 2022.

[CR24] Waters-Davies J (2022). Exploring the impact of the Covid -19 pandemic on learners in Wales.

[CR25] Welsh Government. (2017). *Education in Wales: Our national mission.* Retrieved from https://gov.wales/sites/default/files/publications/2018-03/education-in-wales-our-national-mission.pdf. Accessed 7 Sept 2022.

